# Zika virus outbreak in Suriname, a report based on laboratory surveillance data

**DOI:** 10.1371/currents.outbreaks.ff0f6190d5431c2a2e824255eaeaf339

**Published:** 2018-05-10

**Authors:** John Codrington, Jimmy Roosblad, Amrish Baidjoe, Natanael Holband, Antoine Adde, Mirdad Kazanji, Claude Flamand

**Affiliations:** Academic Hospital, Paramaribo, Suriname; Academich Hospital Paramaribo, Clinical Chemistry, Paramaribo, Suriname; Institut Pasteur and EUPHEM fellow at European Centre of Disease Prevention and Control, Institut Pasteur, Paris, France and European Programme for Public Health Microbiology Training (EUPHEM), European Centre of Disease Prevention and Control (ECDC), Stockholm, Sweden; Academic Hospital, Paramaribo, Suriname; Faculty of Forestry, Geography and Geomatics, Department of Wood and Forest Sciences, Laval University, Québec, Québec, Canada; Institut Pasteur de la Guyane, Cayenne, French Guiana; Epidemiology unitInstitut Pasteur de la Guyane

## Abstract

Introduction : Since the identification of ZIKV in Brazil in May 2015, the virus has spread extensively throughout the Americas. Cases of ZIKV infection have been reported in Suriname since October 2, 2015.  Methods : A laboratory-based surveillance system was quickly implemented according to previous experience with the emergence of chikungunya. General practitioners and public health centers located in different districts of Suriname were asked to send blood samples from suspicious cases to Academic Hospital for molecular diagnosis of Zika virus infection. We investigated Zika-related laboratory data collected during surveillance and response activities to provide the first outbreak report in Suriname in terms of time, location and person. Results : A total of 791 molecularly confirmed cases were reported during a 48-week interval from October 2015 to August 2016. The majority of ZIKV-positive cases involved women between 20 and 39 years of age, reflecting concern about Zika infection during pregnancy. The outbreak peaked in mid-January and gradually spread from the district of Paramaribo to western coastal areas. Discussion : This report provides a simple and comprehensive description of the outbreak in Suriname and demonstrates the utility of laboratory data to highlight the spatiotemporal dynamics of the outbreak in that country.

## Introduction

Zika virus (ZIKV) is a mosquito-borne flavivirus that is closely related to yellow fever and dengue viruses and can be transmitted by the bite of an infected Aedes aegypti mosquito, through sexual contact 1,2,^3^ or from mother to fetus 4 . The first large outbreaks, were not reported until 2007 from the Island of Yap in Micronesia and in May 2015^5^, the World Health Organization reported the first local transmission of ZIKV in the north east of Brazil^6^. Since this initial detection, this virus has spread extensively throughout the Americas6,7,8,9,10,11,12,^13^.

Although ZIKV infections have not historically been regarded as a significant public health concern, during this recent emergence, the virus has been linked to neurological disorders and severe congenital abnormalities. As at July 2017, 48 countries and territories have confirmed autochthonous, vector-borne transmission of ZIKV disease, while five countries have reported sexually transmitted Zika cases. Cases of ZIKV infection have been reported in Suriname since October 2, 2015; this nation, which has 530,000 inhabitants, was one of the first South American countries to report Zika virus infections, after Brazil and Colombia.

Phylogenetic analysis has indicated that the isolated virus belongs to the Asian genotype and appears to be most closely related to a strain that was circulating in French Polynesia in 2013.

This article describes the laboratory based surveillance system in Suriname and the incidence of confirmed cases of ZIKV infection according to sex, age, and spatial and temporal distribution

## Methods

General practitioners and public health centers located in different districts of Suriname were asked to send blood samples from suspicious cases to Academic Hospital for molecular diagnosis of Zika virus infection. In particular, in cases involving pregnant women, samples were screened for free upon request. Viral RNA was extracted from blood and urine samples using a RNeasy Mini Kit (Qiagen, Hilden, Germany). An in-house molecular real-time RT-PCR assessment based on an approach detailed by Lanciotti et al.^14^ was used to confirm all cases. Records from patients with positive results between October 2, 2015, and August 23, 2016, were extracted into a database for epidemiological analyses. Collected data included the date of sample collection, which was used as a proxy for symptom onset; age; gender; and the patient’s district of residence, which was used as a proxy for location. Clinical cases reported by the Suriname Ministry of Health to Pan American Health Organization were compared to the confirmed cases. A clinical case of ZIKV disease was defined as a person with a rash with at least two of the following symptoms: fever higher than 38°C, conjunctivitis (non purulent/hyperemic), arthralgia, myalgia and peri-articular edema.

This analysis is based on data collected during the surveillance and response activities implemented during the ZIKV outbreak in Suriname. All data used in this report were aggregated so that they could not be associated with any specific individual.

## Results

A total of 3,502 samples (2,752 blood samples and 750 urine samples) were collected from 3,460 individuals, and laboratory evidence of ZIKV infection was found in 791 cases. The outbreak spread rapidly throughout the country, reaching all 10 different districts in Suriname. The peak of the epidemic was observed in mid-January (W2016-03), with 107 molecularly confirmed cases (Figure 1). The weekly number of confirmed cases followed the same dynamic as clinical cases reported by the Ministry of Health.

**Temporal and spatial distribution of ZIKV cases in Suriname, October 2015-August 2016:**
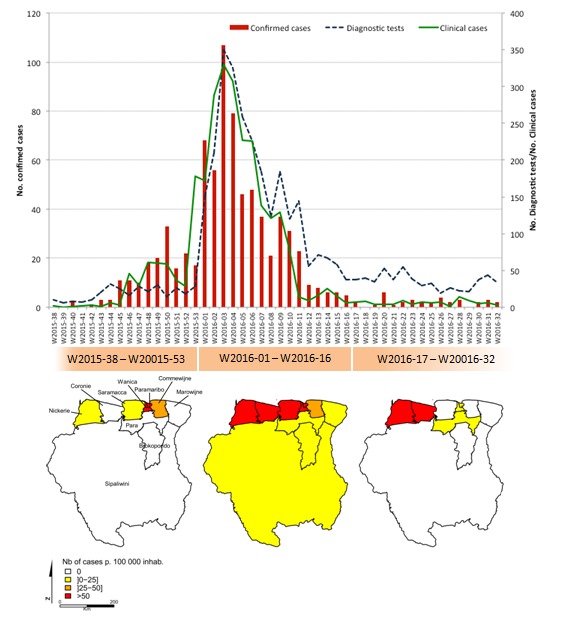

Overall, 69.9% (553/791) of ZIKV-positive cases involved women (Table 1).

**Table 1:** Characteristics of 791 patients with molecular confirmation of Zika virus in Surinam, October 2015 - August 2016.

Characteristics	N	(%)	Incidence per 100,000 population
Sex			
Female	553	(69.9)	85.1
Male	238	(30.1)	201.7
Age group			
<20 years old	111	(13.7)	55.6
[20-39]	413	(51.2)	242.9
[40-59]	190	(23.6)	160.6
>59 years old	93	(11.5)	147.4
District			
Brokopondo	1	(0.2)	12.0
Commewijne	19	(3.3)	60.5
Coronie	5	(8.8)	147.4
Marowoijne	4	(0.7)	19.7
Nickerie	39	(6.9)	113.9
Para	6	(1.1)	24.3
Paramaribo	358	(63.0)	148.6
Saramacca	11	(1.9)	62.9
Sipaliwini	4	(0.7)	10.8
Wanica	121	(21.3)	102.3
Total in Surinam	791		147.7

The majority of ZIKV-positive cases involved patients between 20 and 39 years of age, who accounted for more than 51.2% of cases, reflecting concern about Zika infection during pregnancy that led to the overrepresentation of this population among tested individuals. Small fractions of cases involved patients between 0 and 19 years of age (13.7%) and patients older than 60 years of age (11.5%). More than 60% (358/791) of ZIKV-positive cases were from the district of Paramaribo, which was the first district to report confirmed cases, representing an incidence rate of 1.48 confirmed cases for every 1,000 inhabitants (vs 0.71 for every 1,000 inhabitants in other districts). From October 2, 2015, to November 11, 2015, 11 confirmed cases were reported, all of which were within this district. The outbreak gradually spread to western coastal areas and remained active for an extended period in the western districts of Nickerie and Coronie.

## Discussion

A laboratory based surveillance system for ZIKV infections was quickly implemented in Suriname according to the previous experiences with chikungunya and has monitored ZIKV outbreak dynamics in the different territories of the country.

The Zika virus outbreak represents a major public health threat, particularly for fetuses of infected pregnant women. Even if the aforementioned statistics substantially underestimate the total impact of the outbreak in Suriname because they do not account for unreported clinical illnesses or asymptomatic infections, this report provides a simple and comprehensive description of the outbreak in Suriname and demonstrates the utility of Zika-related laboratory data to highlight the spatiotemporal dynamics of the outbreak in that country. Although the number of involved pregnant women included in the cohort is not available, follow-up of pregnant women who were infected during the outbreak will be critical for improving understanding regarding the spectrum of adverse pregnancy and infant outcomes associated with Zika virus infection and identifying the effects of certain factors, such as the timing of infection during pregnancy.

This report confirms that the timely and passive routine reporting of spatiotemporal information from clinical and laboratory data is critical for determining and communicating infection risks and for implementing risk reduction activities in high-risk areas, especially in the context of a new emerging infectious disease. To adapt prevention messages and activities and improve knowledge, it is essential to rely on a representative multisource surveillance system based on clinical and confirmed cases with particular attention to complications related to neurological disorders, congenital abnormalities and children born from infected mothers.

## Competing Interest Statement

Dr. C. Flamand, on behalf of all the authors of the manuscript submitted to PLoS Current Outbreaks declare that no competing interests exist.

## Data Availability Statement

Weekly laboratory data used in the article are accessible in Supplementary file S1, representing the minimal dataset publicly available. Since de-identified data in this report will not constitute truly anonymous information considering that in some situations (Date of collection, municipalities, sex, age), it could be possible to subsequently link the de-identified data back to an identifiable individual, access to individual data is restricted. Interested researchers may send requests to the head of laboratory of Academic Hospital in Paramaribo, Dr. John Codrington (johncodrington@hotmail.com) Laboratory Director Academic Hospital, Flustraat #1 P.O. Box 9305 Paramaribo, Suriname ; Tel : 011-597-442222

**Supplementary file S1:** Zika virus laboratory data, Academic Hospital-Paramaribo, October 2015 - August 2016.

Epi-week	Confirmed cases	Diagnosis tests
W2015-38	0	10
W2015-39	0	6
W2015-40	2	8
W2015-41	0	7
W2015-42	1	10
W2015-43	3	20
W2015-44	3	31
W2015-45	11	25
W2015-46	11	16
W2015-47	10	28
W2015-48	18	21
W2015-49	20	30
W2015-50	33	34
W2015-51	16	26
W2015-52	22	28
W2015-53	17	28
W2015-01	68	152
W2016-02	56	210
W2016-03	107	350
W2016-04	79	326
W2016-05	46	259
W2016-06	48	226
W2016-07	37	184
W2016-08	21	123
W2016-09	37	184
W2016-10	31	120
W2016-11	23	145
W2016-12	9	56
W2016-13	8	71
W2016-14	6	67
W2016-15	6	58
W2016-16	5	38
W2016-17	2	38
W2016-18	0	40
W2016-19	1	35
W2016-20	6	53
W2016-21	1	38
W2016-22	2	55
W2016-23	3	39
W2016-24	3	29
W2016-25	2	32
W2016-26	4	19
W2016-27	2	26
W2016-28	3	22
W2016-29	1	21
W2016-30	2	38
W2016-31	3	44
W2016-32	2	34

## Corresponding Author

Claude Flamand: cflamand@pasteur-cayenne.fr

## References

[1] Foy BD, Kobylinski KC, Chilson Foy JL, Blitvich BJ, Travassos da Rosa A, Haddow AD, Lanciotti RS, Tesh RB. Probable non-vector-borne transmission of Zika virus, Colorado, USA. Emerg Infect Dis. 2011 May;17(5):880-2. PubMed PMID:21529401. 21529401 10.3201/eid1705.101939 PMC3321795

[2] Hills SL, Russell K, Hennessey M, Williams C, Oster AM, Fischer M, Mead P. Transmission of Zika Virus Through Sexual Contact with Travelers to Areas of Ongoing Transmission - Continental United States, 2016. MMWR Morb Mortal Wkly Rep. 2016 Mar 4;65(8):215-6. PubMed PMID:26937739. 26937739 10.15585/mmwr.mm6508e2

[3] D'Ortenzio E, Matheron S, Yazdanpanah Y, de Lamballerie X, Hubert B, Piorkowski G, Maquart M, Descamps D, Damond F, Leparc-Goffart I. Evidence of Sexual Transmission of Zika Virus. N Engl J Med. 2016 Jun 2;374(22):2195-8. PubMed PMID:27074370. 27074370 10.1056/NEJMc1604449

[4] Calvet G, Aguiar RS, Melo AS, Sampaio SA, de Filippis I, Fabri A, Araujo ES, de Sequeira PC, de Mendonça MC, de Oliveira L, Tschoeke DA, Schrago CG, Thompson FL, Brasil P, Dos Santos FB, Nogueira RM, Tanuri A, de Filippis AM. Detection and sequencing of Zika virus from amniotic fluid of fetuses with microcephaly in Brazil: a case study. Lancet Infect Dis. 2016 Jun;16(6):653-60. PubMed PMID:26897108. 26897108 10.1016/S1473-3099(16)00095-5

[5] Duffy MR, Chen TH, Hancock WT, Powers AM, Kool JL, Lanciotti RS, Pretrick M, Marfel M, Holzbauer S, Dubray C, Guillaumot L, Griggs A, Bel M, Lambert AJ, Laven J, Kosoy O, Panella A, Biggerstaff BJ, Fischer M, Hayes EB. Zika virus outbreak on Yap Island, Federated States of Micronesia. N Engl J Med. 2009 Jun 11;360(24):2536-43. PubMed PMID:19516034. 19516034 10.1056/NEJMoa0805715

[6] Zanluca C, Melo VC, Mosimann AL, Santos GI, Santos CN, Luz K. First report of autochthonous transmission of Zika virus in Brazil. Mem Inst Oswaldo Cruz. 2015 Jun;110(4):569-72. PubMed PMID:26061233. 26061233 10.1590/0074-02760150192 PMC4501423

[7] Enfissi A, Codrington J, Roosblad J, Kazanji M, Rousset D. Zika virus genome from the Americas. Lancet. 2016 Jan 16;387(10015):227-8. PubMed PMID:26775124. 26775124 10.1016/S0140-6736(16)00003-9

[8] Faria NR, Azevedo RDSDS, Kraemer MUG, Souza R, Cunha MS, Hill SC, Thézé J, Bonsall MB, Bowden TA, Rissanen I, Rocco IM, Nogueira JS, Maeda AY, Vasami FGDS, Macedo FLL, Suzuki A, Rodrigues SG, Cruz ACR, Nunes BT, Medeiros DBA, Rodrigues DSG, Queiroz ALN, da Silva EVP, Henriques DF, da Rosa EST, de Oliveira CS, Martins LC, Vasconcelos HB, Casseb LMN, Simith DB, Messina JP, Abade L, Lourenço J, Alcantara LCJ, de Lima MM, Giovanetti M, Hay SI, de Oliveira RS, Lemos PDS, de Oliveira LF, de Lima CPS, da Silva SP, de Vasconcelos JM, Franco L, Cardoso JF, Vianez-Júnior JLDSG, Mir D, Bello G, Delatorre E, Khan K, Creatore M, Coelho GE, de Oliveira WK, Tesh R, Pybus OG, Nunes MRT, Vasconcelos PFC. Zika virus in the Americas: Early epidemiological and genetic findings. Science. 2016 Apr 15;352(6283):345-349. PubMed PMID:27013429. 27013429 10.1126/science.aaf5036 PMC4918795

[9] Pacheco O, Beltrán M, Nelson CA, Valencia D, Tolosa N, Farr SL, Padilla AV, Tong VT, Cuevas EL, Espinosa-Bode A, Pardo L, Rico A, Reefhuis J, González M, Mercado M, Chaparro P, Martínez Duran M, Rao CY, Muñoz MM, Powers AM, Cuéllar C, Helfand R, Huguett C, Jamieson DJ, Honein MA, Ospina Martínez ML. Zika Virus Disease in Colombia - Preliminary Report. N Engl J Med. 2016 Jun 15. PubMed PMID:27305043. 27305043

[10] Lazear HM, Stringer EM, de Silva AM. The Emerging Zika Virus Epidemic in the Americas: Research Priorities. JAMA. 2016 May 10;315(18):1945-6. PubMed PMID:26963564. 26963564 10.1001/jama.2016.2899

[11] Daudens-Vaysse E, Ledrans M, Gay N, Ardillon V, Cassadou S, Najioullah F, Leparc-Goffart I, Rousset D, Herrmann C, Cesaire R, Maquart M, Flusin O, Matheus S, Huc-Anaïs P, Jaubert J, Criquet-Hayot A, Hoen B, Djossou F, Locatelli-Jouans C, Blateau A, McKenzie AM, Melin M, Saint-Martin P, Dorléans F, Suivant C, Carvalho L, Petit-Sinturel M, Andrieu A, Noël H, Septfons A, Gallay A, Paty MC, Filleul L, Cabié A. Zika emergence in the French Territories of America and description of first confirmed cases of Zika virus infection on Martinique, November 2015 to February 2016. Euro Surveill. 2016 Jul 14;21(28). PubMed PMID:27447300. 27447300 10.2807/1560-7917.ES.2016.21.28.30285

[12] Fauci AS, Morens DM. Zika Virus in the Americas--Yet Another Arbovirus Threat. N Engl J Med. 2016 Feb 18;374(7):601-4. PubMed PMID:26761185. 26761185 10.1056/NEJMp1600297

[13] Flamand C, Fritzell C, Matheus S, Dueymes M, Carles G, Favre A, Enfissi A, Adde A, Demar M, Kazanji M, Cauchemez S, Rousset D. The proportion of asymptomatic infections and spectrum of disease among pregnant women infected by Zika virus: systematic monitoring in French Guiana, 2016. Euro Surveill. 2017 Nov;22(44). PubMed PMID:29113627. 29113627 10.2807/1560-7917.ES.2017.22.44.17-00102 PMC5710134

[14] Lanciotti RS, Kosoy OL, Laven JJ, Velez JO, Lambert AJ, Johnson AJ, Stanfield SM, Duffy MR. Genetic and serologic properties of Zika virus associated with an epidemic, Yap State, Micronesia, 2007. Emerg Infect Dis. 2008 Aug;14(8):1232-9. PubMed PMID:18680646. 18680646 10.3201/eid1408.080287 PMC2600394

